# Lead accumulation and elimination in tissues of Prussian carp, *Carassius gibelio* (Bloch, 1782), after long-term dietary exposure, and depuration periods

**DOI:** 10.1007/s11356-012-1210-8

**Published:** 2012-10-06

**Authors:** Ewa Łuszczek-Trojnar, Ewa Drąg-Kozak, Włodzimierz Popek

**Affiliations:** Department of Ichthyobiology and Fisheries, University of Agriculture in Krakow, Spiczakowa 6, 30-199 Kraków-Mydlniki, Poland

**Keywords:** Prussian carp, Dietary, Lead, Exposure, Accumulation, Depuration

## Abstract

We studied the bioaccumulation of lead in selected tissues of Prussian carp *Carassius gibelio* (Bloch, 1782) during 12 and 24 months exposure to different doses of this metal in feed and the elimination of lead from tissues during the following 12-month depuration period. Lead concentration was determined using atomic absorption spectrometry method. The highest lead concentrations were observed at 2.0 ± 0.54 to 7.4 ± 1.1 mg kg^−1^ in the kidney, 3.0 ± 0.13 to 5.2 ± 0.17 mg kg^−1^ in the bone, and 4.5 (±0.4) mg kg^−1^ in the hepatopancreas of fish from groups exposed to lead dietary concentration from 8 to 49 mg kg^−1^ for 24 months. The rate of accumulation were generally the highest at the beginning of exposure as evidenced by the highest monthly increments of bioaccumulation observed after 3 months of contamination for muscles, hepatopancreatic gland, intestine, and gills. Also analysis of the monthly increments of lead bioaccumulation in bone tissue and the highly significant coefficients of correlation indicate that the dynamics of accumulation are clearly dependent on dose of exposure. Depuration of accumulated lead from the organs depended mainly on tissue and duration of elimination period. Very rapid depuration was observed in soft tissues such as the intestine or muscles. Very low elimination was observed for scales and bones where until the end of the experiment highly significant lead concentration differences were observed in all groups in relation to the control group. Chronic dietary exposure in the range of 8–49 mg Pb kg^−1^ resulted in no significant effects on the growth and survival of Prussian carp females.

**Electronic supplementary material:**

The online version of this article (doi:10.1007/s11356-012-1210-8) contains supplementary material, which is available to authorized users.

## Introduction

Lead belongs to a group of toxic metals which have no function in the physiological processes of living organisms (Adeyeye et al. 1996). Limit values of lead according to Polish standards average 7.2 μg l^−1^ for surface waters (Journal of Laws 2011) and 200 mg kg^−1^ for bottom sediments (Journal of Laws 2002). Reports from 2009 for the largest Polish river, the Vistula, and its tributaries, showed that despite the fact that lead levels in water were not exceeded, high concentrations of this metal (in excess of 1,800 mg kg^−1^) were observed in bottom sediments (GIOS 2010). It is well known that this metal easily accumulates in fish tissues such as bones, gills, kidneys, liver, and scales (Dallas and Day 1993). Because lead crosses the blood–brain barrier, it can cause various detrimental effects to the body condition, health, and life of fish (Hodson et al. 1978; Needleman and Bellinger 1991; Rabitto et al. 2005). Bioaccumulation of lead in piscine tissues is a threat to humans who may absorb considerable amounts of this metal from contaminated fish. It is common knowledge that lead adversely affects the intellectual development of children, increases arterial pressure, and contributes to cardiovascular diseases in adults (EC 2001). Therefore, the importance of studies of lead bioaccumulation in fish, which represent a valuable source of food for humans, in the context of environmental pollution, seems unquestionable. Heavy metals enter the body of fish through the skin or gills via the dissolved phase and through the digestive tract via the food (Pourang 1995; Vincent et al. 2002; Sarnowski 2003). Because waters generally have low lead levels, even when high concentrations are found on the bottom, food is a significant source of these elements for fish (Javed and Hayat 1996; Clearwater et al. 2000). Therefore, the aim of the present study was to investigate the bioaccumulation of lead in selected tissues of Prussian carp during long-term exposure (12 and 24 months) to different doses of this metal in feed and the elimination of lead from tissues during the following 12-month depuration period.

The distribution of metals (Hg, Cd, Pb, Cr, Cu, Zn, Ni, Fe, and Mn) in the tissues of different fish species such as perch, roach, chub, white bream, smelt, grayling, or pike was described previously, with fish used as bioindicators of water pollution (Spurný et al. 2002; Staniskiene et al. 2006). The Prussian carp was chosen as an experimental model due to its low sensitivity and high resistance to adverse environmental conditions. The Prussian carp is an important subject for model experiments involving heavy metals, biomonitoring, and industrial and amateur fishing (Syasina et al. 2012). This species is widely distributed in Europe, Siberia, and Asia; in the eastern regions, it is represented by bisexual populations with single monosex polyploid populations (Fan and Liu 1990). Most European populations are monosex populations, living as triploid females that reproduce gynogenetically (Boroń et al. 2011), and such a population was chosen for this study to eliminate the effect of sex on the accumulation or elimination of lead during the experiment.

The dietary lead levels to which Prussian carp were exposed in the present experiment were based on the reported concentrations of this metal in benthic organisms that are the Prussian carp’s natural food, which may range from 0 to as much as 792 μg Pb g^−1^ dry weight (dw; Hodson et al. 1978; Woodward et al. 1994). Given that the study was aimed to show the effect of long-term exposure (the normal level of lead in invertebrates in an unpolluted environment should not exceed 1 μg g^−1^ (Farag et al. 1999)), we chose concentrations simulating the average degree of environmental contamination (8, 13, 24, and 49 mg kg^−1^) to find out if their constant presence in fish diets will significantly affect the bioaccumulation and subsequent elimination of lead from Prussian carp tissues, and whether the bioaccumulation will vary according to the dose of exposure. An attempt was also made to observe the dynamics of the bioaccumulation and elimination processes depending on duration of exposure and depuration.

## Materials and methods

### Fish and diet

Used in the study are 420 female Prussian carps *Carassius gibelio* (Bloch, 1782), originating from “Górki” Fish Farm in Wiślica, Poland. Fish were stocked in fourteen 300-l aquaria (30 fish per aquarium) at water supplied from the Municipal Waterworks in Cracow, an average temperature of 17 °C, pH of 7.7, dissolved oxygen of 10.5 mg l^−1^, and water hardness of 127 mg CaCO_3_ l^−1^. In addition, the water was aerated and filtered. The experiment was preceded by a 4-month adaptation period when fish were fed a control diet (lead concentration of 0.1 mg kg^−1^ dw). Exposure to lead began when fish were 9 months old, had an average body weight of 20 g and body length of 10 cm. Fish were divided into five groups which were fed control pellets (group 1) and pellets contaminated with different concentrations of lead (8, 13, 24, and 49 mg Pb kg^−1^—groups 2–5) for two exposure periods of 12 and 24 months (Table 1). Pellets of complete feed for Prussian carp were produced by the Institute of Ichthyobiology and Aquaculture of the Polish Academy of Sciences in Gołysz using the components: grains, oilseed, fish meal, and vitamin and mineral supplements. Specifications for the fodder were 37 % crude protein, 12 % crude fat, and 31 % carbohydrates. Control and experimental pellets were prepared the same way, but there were no Pb addition into the control fodder. Lead acetate trihydrate Pb(CH_3_COO)_2_^.^ 3H_2_O (POCh S.A.) was added in appropriate proportions into standard ingredients before the pelletizing during the production of pellets process. Analysis of lead concentrations in pellet samples showed the following mean levels: group 1 control, 0.113 (±0.03) mg kg^−1^; group 2, 8.07 (±0.11) mg kg^−1^; group 3, 13.11 (±0.14) mg kg^−1^; group 4, 23.71 (±0.18) mg kg^−1^; and group 5, 48.62 (±0.24) mg kg^−1^ dw.

**Table 1:** The configuration of treatment groups and lead doses in diet of fish during the 12 months exposure period and the following 12 months exposure or depuration period

Groups	1–control	2	2-dep	3	3-dep	4	4-dep	5	5-dep	total
Pb dose in diet (mg kg^−1^) during the first 12 months period	0.1	8		13		24		49		-
Number of fish at the beginning [*n*] (per aquarium)	30/30	30/30/30		30/30/30		30/30/30		30/30/30		420
Number of fish after 3 months [*n*]	26/26	28/27/27		28/27/27		28/27/27		28/27/27		380
Number of fish after 6 months [*n*]	20/20	24/25/24		25/25/24		25/25/24		25/25/24		335
Number of fish after 12 months exposure	14/14	13/14	15/15	14/14	15/14	14/14	15/15	13/14	15/15	257
Pb dose in diet [mg kg^−1^] during the second 12 months period	0.1	8	0.1	13	0.1	24	0.1	49	0.1	–
Number of fish after 15 months [*n*]	10/10	9/10	11/11	10/10	11/10	10/10	10/11	9/10	11/11	184
Number of fish after 18 months [*n*]	5/4	5/5	5/5	5/5	6/5	5/5	4/5	5/5	5/4	88

After 12 months of exposure, each experimental group was divided into two groups of fish, one of them were under the same treatment and the second (groups: 2-dep, 3-dep, 4-dep, and 5-dep) experienced a depuration period of 12 months and received the control feed until the end of the experiment (Table 1). Throughout the study, fish were fed once daily a ration that amounted to 3 % of their body weight. Feed intake was recorded.

To avoid repeated exposure of fish to the intake of excreted lead from water, aquarium water was changed every 2 weeks. Prior to the first three water changes, water and bottom sediments (excrements and food remains) Pb concentration was analyzed.

After 12 months of the experiment, fish females reached sexual maturity. Water temperature was raised to 20 °C; after the third day, fish presented spawning behavior. Despite the absence of males, fish ovulated and spawned. Unfertilized eggs were observed in aquariums of all groups.

Fish of each aquarium were individually weighted before exposure and after 12 and 24 months of experiment to study effect of different Pb dietary doses to body weight.

### Tissue sampling

At 3, 6, 12, 15, 18, and 24 months of the experiment, the following tissues were collected from 8 to 10 randomly harvested fish from each group: kidneys, gills, intestine (divided into anterior and posterior intestine, both without digesta, rinsed with deionized water), muscles, hepatopancreatic gland, skin, scales, and bone tissue from opercula freed from skin. These tissues were frozen until analysis of lead concentration.

### Pb analysis

Prior to determination, tissues (of weight from 1 to 5 g) were subjected to preliminary mineralization in presence of 10 ml a 3:1 *v/v* mixture of nitric acid (65 %) and perchloric acid (70 %) for about 20 h. The samples were then heated with a Velp 20/26 digester by gradually increasing the temperature to 180 °C for 6–7 h. The so obtained clear liquid was diluted with deionized water to 10 ml and then assayed for concentration of lead using a Unicam 929 atomic absorption spectrometer (Agemian et al. 1980). The concentrations were read from the standard curve generated using the standards prepared based on atomic absorption standards made at the Office of Weights and Measures in Warsaw. Each sample was assayed in quadruplicate (the average values calculated from replicates were used in statistical analysis). Calibration was repeated every 10 samples. The results were presented in mg Pb per kg wet tissue weight (ww).

Water samples of 5 ml volume, collected from aquaria of different groups, were mineralized in the presence of nitric acid (10 ml) at 150 °C for 4 h, and then diluted to 10 ml with deionized water (Ihnat 2003). So prepared samples were analyzed for the level of Pb in the same way as tissue samples.

Bottom sediment samples were dried in the temperature of 60 °C for 48 h (Twyman 2005). Samples of 3 g dw were mineralized in the presence of 10 ml nitric acid at room temperature (20 °C) for 20 h and at 150 °C for 8 h, and then diluted to 10 ml with deionized water; Pb level was determined as described above. The results were presented in milligrams Pb per kilogram dw.

Random pellet samples (weight, 3 g) were mineralized in the presence of nitric acid (10 ml) for about 20 h at room temperature and then for about 7 h at 150 °C. Finally, the samples were diluted with deionized water to 10 ml and the level of Pb was determined as described above. The results were presented in milligrams Pb per kilogram dw. Minimum level of detection for lead was 0.001 mg l^−1^ of water samples and 0.001 mg kg^−1^ of solid samples.

### Statistical analysis

The results were subjected to analysis of variance and the Mann–Whitney test was used to determine significant differences between the experimental and control groups, between the experimental groups (2, 3, 4 and 5) and between the depurated groups (2-dep, 3-dep, 4-dep, 5-dep) within each sampling, and between following samplings within the same group. Spearman’s correlation coefficients were also calculated to determine significance of the effect of lead dose during exposure on its concentration in individual tissues.

### Dynamics of accumulation or depuration

Because samples were collected at varying time intervals (every 3 or 6 months) to observe the dynamics of accumulation or depuration, monthly increments of lead concentration and monthly lead depuration rate were calculated for appropriate periods by dividing total increment of accumulation calculated for 3, 6, 12, 15, 18, and 24 months of exposure—after which the tissues were sampled, into appropriate number of months for each of these periods. This produced mean monthly increments of Pb bioaccumulation in tissue in corresponding exposure periods (metal level end of metal accumulation − metal level before exposure)/months of metal exposure.

Monthly metal depuration rate was calculated by dividing the difference in the mean lead concentration at the end of exposure period and the concentration after the elimination period by the number of depuration months 3, 6, and 12; (metal level end of metal accumulation − metal level end of metal depuration)/months of metal depuration. The method for calculating the dynamics of lead accumulation and elimination adapted from Yap et al. (2003).

## Results

The lowest mean body weight (27.1 ± 4 g) was observed in fish of group 3 after a 12-month period of Pb exposure to 13 mg Pb kg^−1^, while the highest body weight 40.4 g occurred in the control group. After 24 months of the experiment, mean body weight ranged from 34 ± 7 g in fish of group 5-dep to 74 ± 20 g in group 2, where fish females were exposed to 8 mg Pb kg^−1^. Chronic dietary exposure in the range of 8–49 mg Pb kg^−1^ resulted in no significant effects on the growth of Prussian carp females (Table 2). The total mortality rate was equal to nine pieces (Table 2).

**Table 2:** Body weight changes after 12 and 24 months of the experiment, and total mortality of fish

Groups	1–Control	2	2-dep	3	3-dep	4	4-dep	5	5-dep	total
Mean body weight [g] of fish at the beginning of exposure	19.1 ± 2 a	19.7 ± 2 a		20.1 ± 3 a		20.8 ± 2 a		19.9 ± 2 a		-
Mean body weight [g] of fish after 12 months of exposure	40.4 ± 6 a	36.2 ± 4 a		30.9 ± 4 a		27.1 ± 4 a		30.6 ± 6 a		-
Mean body weight [g] of fish after 24 months of exposure	51.2 ± 8 ab	74 ± 20 a	67 ± 11 a	44 ± 5 ab	65 ± 8 a	48 ± 12 ab	51 ± 19 ab	53 ± 13 a	34 ± 7 b	-
Total mortality [fish]	2	1	0	1	1	0	2	1	1	9

Different letters indicate statistically significant differences among fish groups at *p* < 0.05 (Mann–Whitney *U* test). Values not sharing letters are significantly different from other treatment values within the same sampling

Lead concentration in water samples collected prior to biweekly water replacement was under the spectrometer limit detection (0.001 mg Pb L^−1^). Mean lead concentration in bottom sediments of aquariums in groups 1–5 were amounted to 1.54 (±0.2), 13.74 (±0.8), 24.21 (±2.3), 30.88 (±3.0), and 71.80 (±4.4) mg Pb kg^−1^ dw, respectively.

**Fig. 1:**
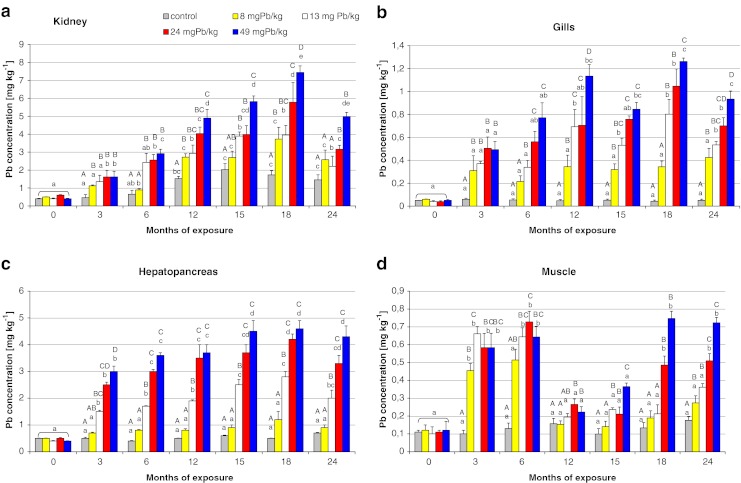
The comparison of mean lead concentration (milligrams per kilogram tissue wet weight) in the kidney, gills, hepatopancreas, and muscle of Prussian carp females during 24-month period dietary exposure to different levels of lead in the diet. *Different letters* indicate statistically significant differences among groups at *p* < 0.05 (Mann–Whitney *U* test). *Values not sharing uppercase letters* are significantly different from other treatment values within the same sampling. *Values not sharing lowercase letters* are significantly different from the same treatment between months. Note the different *y*-axis scale

**Fig. 2:**
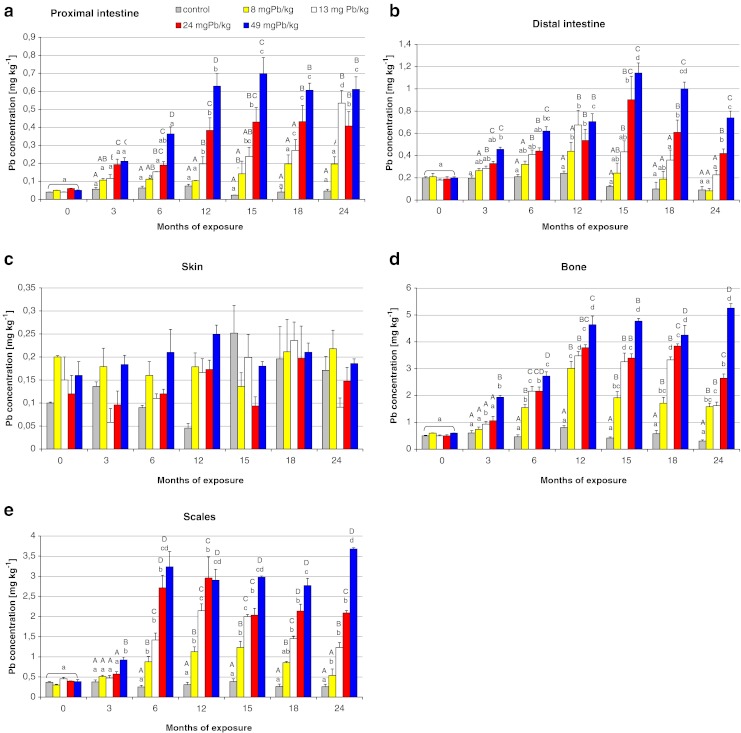
The comparison of mean lead concentration in the proximal and distal intestine, skin, bone, and scales of Prussian carp females during 24-month period dietary exposure to different levels of lead in the diet. There were no significant differences in skin tissue. Other details as in legend of Fig. 1

Figures 1 and 2 show the lead accumulation levels in studied tissues during the 24-month period of dietary exposure. The tissues that accumulated more Pb were kidney, bone, and hepatopancreas, reaching values of 7 (±0.72), 5 (±0.0.43), and 4.5 (±0.4) mg kg^−1^ ww, respectively, after 12–24 months of exposure (Figs. 1a, c and 2d). Statistical significant differences in the lead accumulation levels between groups were observed in all studied tissues (*p* < 0.05) except in the skin (Fig. 2c).

The accumulation of lead was generally the highest at the beginning of exposure, as evidenced by the highest monthly increments of bioaccumulation observed after 3 months of contamination for muscles, hepatopancreatic gland, intestine, and gills (Electronic supplementary material (ESM) Fig. 1S). The dynamics of lead accumulation in the kidney were almost similar throughout the whole exposure period for all doses used. In the case of bone and scales tissues with lower doses of exposure in groups 2, 3, and 4 (and 5 in case of scales) monthly increments of bioaccumulation were highly significant only after 6 months; whereas in group 5, intensive bioaccumulation was already observed during the first 3 months of contamination in the bone (ESM Fig. 2S).

**Fig. 3:**
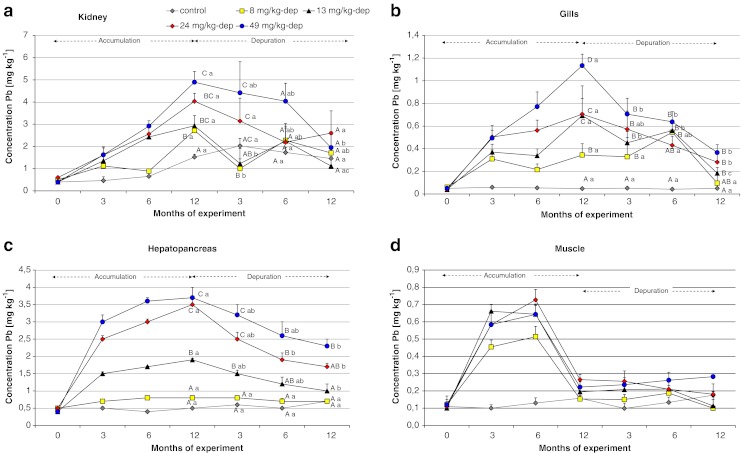
Changes of Pb concentration in the kidney, gills, hepatopancreas, and muscle of Prussian carp females during 12 months dietary exposure to that metal and following 12 months depuration periods. Significant differences of lead concentration between groups (uppercase letters) and in dependence on time depuration (*lowercase letters*). Different letters mean significant difference *p* < 0.05. There were no significant differences in muscle tissue. Note the different *y*-axis scale

**Fig. 4:**
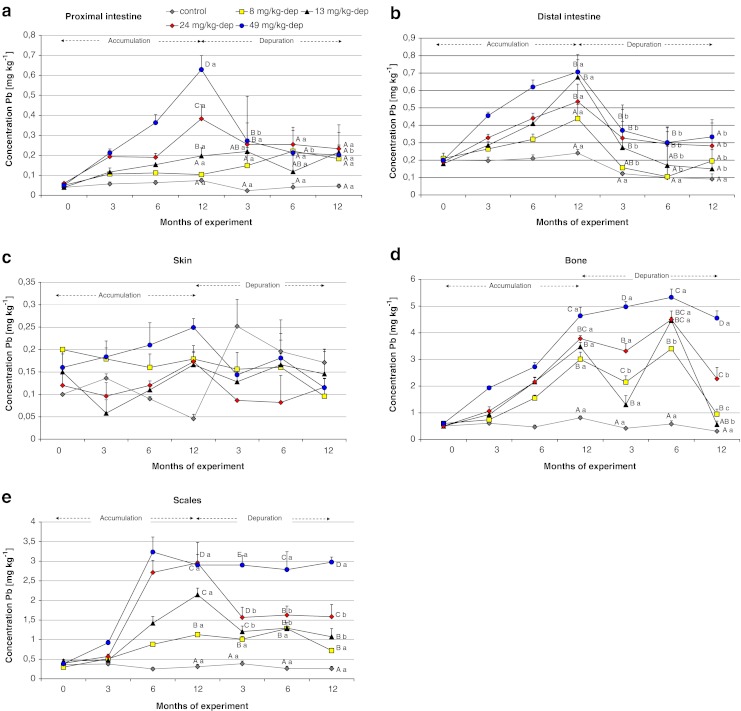
Changes of Pb concentration in the proximal and distal intestine, skin, bone, and scales of Prussian carp females during 12 months dietary exposure to that metal and following 12 months depuration periods. There were no significant differences in skin tissue. Other details as in legend of Fig. 3

The Spearman’s correlation coefficients R analysis results presented in ESM Table 1S show the significance of the lead dose effect on its accumulation in studied tissues. Figures 3 and 4 present changes of lead concentration in studied tissues of Prussian carp during 12 months dietary exposure to that metal and following 12 months of depuration period. The highest monthly decreases in accumulation were observed in the first 3 months of elimination in kidneys (0.57 mg kg^−1^ ww/month in groups 2-dep and 3-dep), in bones (0.72 mg kg^−1^ ww/month in group 3-dep), in scales (0.46 mg kg^−1^ ww/month in group 4-dep), and in hepatopancreas (0.33 mg kg^−1^ ww/month in groups 4-dep and 5-dep; ESM Fig 2S). In soft tissues, higher monthly decreases in concentration were observed in the groups previously exposed to higher doses of exposure (4-dep and 5-dep), whereas in hard tissues, such as bones and scales, accumulation rather than elimination was still observed in group 5-dep after the first 3 months (Fig. 4d, e and ESM Fig. 2S). Until the end of the elimination period, statistically significant differences (*p* < 0.05) were observed between the level of lead in the groups subjected to depuration, both in bone tissue and in scales (Fig. 4d and e).

## Discussion

### Growth and survival

No significant differences were observed in fish body weights after 12 and 24 months of experiment (Table 2). These results are consistent with Alves and Wood (2006), Alves et al. (2006), and Mount et al. (1994) who found no effect of Pb on growth of rainbow trout. Different results noted Woodward et al. (1994) or Naz et al. (2008) who observed significantly lower body weight increments in fish during the heavy metals exposure. The total mortality rate was equal to nine pieces (Table 2) and was not associated with the dietary Pb treatments throughout the course of the experiment what is consistent with other dietary studies results (Hodson et al. 1978; Mount et al. 1994; Alves et al. 2006). Some fish species show higher resistance to heavy metals, what may be based either on their ability to accumulate excessive amounts of metals or to exclude heavy metals from tissues. Growth rates and mortality do not appear to be sensitive indicators of dietary Pb toxicity in Prussian carp.

### Accumulation

The only source of Pb for fish in the present study was the fodder. To avoid the dissolution of Pb from feces and pellets remains, aquarium water was changed every 2 weeks. Water analysis indicated no detectable Pb level (detection limit, 0.001 mg Pb L^−1^) after 2 weeks of feeding fish with the contaminated pellets, despite the Pb concentrations in bottom sediments in aquariums were enhanced, from 1.5 (in control group) to 78 mg Pb kg^−1^ dw (in group 5).

The results obtained suggest that lead bioaccumulation in females of Prussian carp differs in dependence on tissues. The bioaccumulation pattern of lead in different tissues was: kidney > bone > liver > scales > gills > distal intestine > muscle > proximal intestine > skin. Physiological differences in the function and structure of different tissues may affect the bioaccumulation of metals (Kotze 1997). Daily intake of lead ranged from 0.24 to 1.47 mg kg^−1^ ww (body weight)/day depending on the dose of exposure, so the fish received a total dose of 108–540 and 216–1,080 mg Pb kg^−1^ body weight after 12 and 24 months of exposure, respectively. It is known that a major portion of ingested metals do not accumulate in tissues and are excreted after passing through the digestive system (Glover and Hogstrand 2002). Part of lead, which had been absorbed from the digestive tract into the blood, is distributed into different body tissues, where it undergoes bioaccumulation or is removed through the gills, kidneys, or skin. This may be the reason why such high Pb concentrations were noted in the kidney (Fig. 1a). Pb level in the kidney of control fish significantly increased after 12 months of experiment and did not decreased to the end, despite the fish remained in the same conditions as the acclimation period. There was observed not significant decrease of Pb concentration in distal intestine and bone tissues of control fish, that would be accumulated in the kidney, but more likely the enhanced Pb level in the kidney was the effect of slow accumulation of Pb little rates 0.003 mg Pb kg^−1^ ww (body weight)/day, from control pellets which Pb concentration was 0.113 mg kg^−1^ dw. The kidney was the only tissue where the increased Pb level in control group was observed, what indicate that this organ can play the important role in maintain of organism homeostasis.

When observing the process of Pb accumulation during long-term exposure, it is easy to see the dose dependence. In all tissues, the highest accumulation resulted from exposure to the highest dietary dose of lead, as confirmed by high correlation coefficients (from 0.61 to 0.96) for Pb concentration and dose in successive periods of the study (ESM Table 1S). Skin is the only tissue which does not reflect the food Pb contamination and no significant differences occurred in Pb accumulation between control and treatment groups as well as between following samplings (Fig. 2c and ESM Table 1S).

In all studied tissues except for skin, lead concentration increased steadily with increasing exposure time, and after reaching the maximal level periodically decreased despite continued exposure (Figs. 1 and 2). More or less stable Pb levels in tissues suggest that fish may be able to regulate lead in organism. This regulation depends on Pb dose in diet. Lower Pb exposure reflects lower maximal Pb concentration levels in tissue. For example the maximal Pb level of 1.1 mg kg^−1^ was observed after 15 months of exposure to 8 mg Pb kg^−1^ and significant reduction was noted after 24 months in scales. After 12 months of exposure to 13 mg Pb kg^−1^, the maximal Pb concentration 2.2 mg kg^−1^ was noted in the tissue and significant decrease after 18 months. During the exposure to higher Pb doses of 24 and 49 mg kg^−1^, maximal observed Pb concentrations were 2.9 and 3.7 mg kg^−1^, respectively, and no significant decreases during the rest of exposure were noted (Fig. 2e). Similar changes in Pb concentration were observed in bone, kidney, gills, intestine, or hepatopancreas (Figs. 1 and 2). Alves and Wood (2006) noted related Pb accumulation effect during 42 days dietary Pb exposure of juvenile rainbow trout, suggesting that some tissues are able to adjust, regulate, and redistribute Pb to other tissues for detoxification or excretion of Pb. Steady state of Pb concentration observed in all tissues except skin and muscle after 12 months of Pb dietary exposure maybe reflects equilibrium between uptake and elimination.

Pb concentration in muscle tissue was also observed to increase to the maximum level of 0.72 (±0.06) mg kg^−1^ during the first 6 months of exposure to 24 mg Pb kg^−1^ (Fig. 1d). Maximum lead levels for certain contaminants in foodstuffs amount to 0.3 mg kg^−1^ ww (EC 2006), so the concentration values observed during fish exposure in meat exceeded the limit more than two times. Odžak and Zvonaric (1995) noted similar concentration values after 60-day exposure of *Dicentrarchus labrax* to a dose of 238 mg kg^−1^, as well as a clear relationship between accumulation (in both muscles and the liver) and the applied dose of lead exposure. The low accumulation of lead in muscles compared to other piscine tissues is also in agreement with other authors (Bradley and Morris 1986; Wagner and Boman 2003; Jabeen and Chaudhry 2010). A decrease in the level of this metal to the values comparable with control values was observed in muscle tissue between 12 and 15 months of exposure (Fig. 1d). Such depuration of lead from the muscles could be due to fish becoming more resistant in the second year of life, when the development of all body was completed, sexual maturity was reached and the immune system was fully developed. Until the end of exposure, no significant increments of Pb concentration were observed in groups 2 and 3, and there were no differences in relation to the control group despite the fact that in groups 4 and 5, Pb concentration increased again to the values similar to those observed in the first year of exposure, i.e., 0.51 (±0.04) and 0.75(±0.03) mg kg^−1^, respectively (Fig. 1d). It may be that mature fish remained insensitive to lower doses of lead exposure, but the spawning and gonadal restoration again weakened their resistance to higher doses in groups 4 and 5, and Prussian carp responded with reaccumulation of lead in their muscles. Before the next spawning, which occurred at 24 month of the experiment, lead concentration in muscles was not observed to decrease, which excludes the reproductive period as the cause of earlier self-depuration.

For the intestine, higher lead concentrations were observed in the posterior part, where the highest mean concentration of 1.1 (±0.09) mg kg^−1^ occurred after 15 months of exposure to 49 mg Pb kg^−1^ in group 5 (Fig. 2b); whereas in the anterior part of the digestive tract, the highest mean concentration was 0.7 (±0.09) mg kg^−1^ (Fig. 2a). Such distribution of accumulation in the intestine was to be expected because breakdown and digestion take place in the anterior part, whereas the posterior part of intestine in nonpredatory fish is responsible for nutrient absorption, which is why most of the lead was absorbed there. Reverse proportions of lead accumulation in the intestine were reported by Alves and Wood (2006) in trout during 42-day exposure to different combinations of lead and calcium doses. Because of the role it plays in the body, the intestine is particularly at risk of accumulating highest lead concentrations; in contrast, the intestinal levels were comparable to those observed for other soft tissues. This may be due to the protective role of mucus secreted from the intestinal walls, which binds and entraps large amounts of toxic compounds (including lead) from food, thus preventing their absorption by the tissue, which was observed during intestinal perfusion with zinc (Glover and Hogstrand 2002). Crespo et al. (1986) showed that rainbow trout orally administrated with Pb ((10 μg Pb/g dw)/fish/day) had morphological alternations of the intestinal brush border, increased mucous cell activity, an increased renewal rate of absorptive cells.

Liver is regarded as an indicator of environmental contamination, because repeatedly demonstrated the existence of dose dependent effect between levels of pollution in the aquatic environment (Hinton and Lauren 1990; Pesch and Kraus 1991; Stentiford et al. 2003), which is confirmed by the constant increments of bioaccumulation and the rapid depuration of considerable portions of accumulated lead in the first period of elimination (ESM Figs. 1S and 2S). The hepatopancreas accumulates relatively higher amounts of Pb exceeding 4 mg kg^−1^ body weight during the exposure to 24 and 49 mg Pb kg^−1^ (Fig. 1c). According to Heath (1991), fish can regulate metal concentration to a certain limit after which bioaccumulation occurs. For the liver of Prussian carp, it seems to be the dietary dose higher than 8 mg Pb kg^−1^, because no significant changes were noted during 24 months period of the exposure to that dose compared to control group or between the following samplings (Fig. 1c).

Among hard tissues, the highest concentration was found in bones (5.24 ± 0.17 mg kg^−1^ at 24 months of exposure in group 5; Fig. 2d). Such a result was to be expected considering that this tissue is the primary target of lead accumulation (Kurey 1991; Rabinovitz 1991; Sorensen 1991). In the groups of fish exposed to lower doses of lead (8 and 13 mg kg^−1^; groups 2 and 3, respectively), the accumulation of this metal in the bone in the first year of exposure was twice as high as in the second year, and lead concentrations were indeed observed to decrease in groups 2 and 3, possibly indicating that partial self-depuration from the tissue could occur in the second year of exposure to lower Pb concentrations (8–13 mg Pb kg^−1^ dw), despite continued exposure (Fig. 2d). No decreases in lead concentration were observed for higher doses in groups 4 and 5, but it is easily seen that almost all of the accumulated lead was incorporated during the first year of exposure. There were observed no statistical differences in groups between months since the 12th month of experiment (Fig. 2d). Also, analysis of the monthly increments of bioaccumulation in bone tissue (ESM Fig. 1S) and the highly significant coefficients of correlation (ESM Table 1S) indicate that the accumulation is clearly dependent on dose of exposure. While the highest monthly increments of lead are observed after 12 months of exposure in group 2 (0.09( ± 0.001) mg Pb kg^−1^ ww/month) and after 6 months in groups 3 and 4 (0.28 (± 0.008) mg Pb kg^−1^ ww/month), in group 5 they already reach a mean value of 0.44 (± 0.02) mg kg^−1^ ww/month after 3 months (ESM Fig. 1S). This is evidence that the higher the dose of exposure, the quicker the accumulation is in bone tissue. Exactly the same accumulation pattern was observed in scales, where very high coefficients of correlation between Pb concentration and exposure dose were noted (ESM Table 1S). The higher lead concentrations in hard tissues (bones and scales) than in other tissues were also reported by Rashed (2001) for Nile tilapia from Aswan High Dam Lake in Egypt.

### Depuration

Depuration of accumulated lead from the organs during exposure of 12 months depended mainly on tissue and duration of elimination. Very rapid depuration is observed in soft tissues such as the intestine or muscles, where a relatively quick decrease in Pb concentration was observed in the groups subjected to elimination (ESM Fig. 2S) and no statistically significant differences in Pb concentration were found in these groups in relation to the control group (Figs. 3d and 4a, b). In the case of muscles, it is difficult to show whether the decreased Pb level was due to cessation of exposure, because self-depuration was already observed during exposure. Lead elimination from the kidneys, gills, and hepatopancreas was slightly slower, probably due to their role in the removal of this element from the body (Fig. 3a–c). The elevated concentrations of lead during the 12-month depuration period in these tissues may result from secondary accumulation of the metal released from other soft and hard tissues, which managed to accumulate large amounts of lead, and after exposure cessation they responded by releasing accumulated lead into the blood. This is why these tissues still contained lead, although exposure was discontinued a long time before. Effective elimination from the gills and muscles of Nile tilapia was observed after 4-week exposure to lead in water by Al-Nagaawy (2008), who found that elimination from this tissue is different to elimination from the muscle, as well as suggesting that the elimination is related to the earlier dose of exposure.

Very low elimination was observed for scales and bones (Fig. 4d and e) and until the end of the experiment highly significant differences were observed in all the groups in relation to the control group. After occupational exposure, the slow decline in blood lead, a 5- to 19-year half-life, reflects the long skeletal half-life in people (Rabinowitz 1991). Such a low capacity for depuration of hard tissues may be used for monitoring lead contamination of the fish habitat, even a long time after its disappearance. This may make it possible to use these tissues as bioindicators of long-term environmental pollution. Scales collected from live fish can be used for monitoring contamination of fish without killing them, i.e., without loss of ichthyofauna.

## Conclusion

The results obtained from this study indicate that chronic dietary exposure in the range of 8–49 mg Pb kg^−1^ resulted in no significant effects on the growth or survival of Prussian carp females and these parameters do not appear to be sensitive indicators of dietary Pb toxicity in this species.

Pb concentration in all studied tissues except the skin is positively correlated with the metal concentration in the diet. Kidney, bone, and hepatopancreas are the tissues that accumulated more Pb, reaching values of 7 (±0.72), 5 (±0.0.43), and 4.5 (±0.4) mg kg^−1^ ww, respectively, after 12–24 months of exposure. The accumulation of lead was generally the highest at the beginning of exposure. It is accepted that the concentration of pollutants in the tissues of aquatic animals results from both past and recent pollution of the environment (Ravera et al. 2003). The results of the present study show that a simple analysis of lead concentrations in different tissues may reflect recent environmental contamination, when only soft tissues undergo accumulation; long-term contamination, when all soft and hard tissues undergo accumulation; or contaminants no longer found in the environment, when their presence is only detected in hard tissues such as bones and scales. Researchers interested in studying only long-term contamination may limit themselves to sampling scales to monitor the contamination of fish in a way that poses no threat to their health and lives.

## Electronic supplementary material

Below is the link to the electronic supplementary material.

ESM 1 DOC 80.0 kb
