# Supplementary material for: Lead accumulation and elimination in tissues of Prussian carp, Carassius gibelio (Bloch, 1782), after long-term dietary exposure, and depuration periods
Source: Environ Sci Pollut Res Int. 2012 Oct 6;20(5):3122–32. doi: 10.1007/s11356-012-1210-8 (PMC3633783; doi:10.1007/s11356-012-1210-8)
Supplement: Supplementary file 1 — DOC 80.0 kb [file 11356_2012_1210_MOESM1_ESM.doc]

Supplementary material

**Lead accumulation and elimination in tissues of Prussian carp, *Carassius gibelio* (Bloch, 1782), after long term dietary exposure and depuration periods**

*Ewa Łuszczek-Trojnar ,Ewa Drąg-Kozak, Włodzimierz Popek*

Department of Ichthyobiology and Fisheries, University of Agriculture in Krakow
Author’s address: dr Ewa Łuszczek-Trojnar – corresponding author
dr Ewa Drąg-Kozak, prof dr hab. Włodzimierz Popek
Department of Ichthyobiology and Fisheries,
University of Agriculture in Krakow, Spiczakowa 6,
30-199 Kraków-Mydlniki, Poland

e-mail: [e.trojnar@ur.krakow.pl](mailto:e.trojnar@ur.krakow.pl)

tel 48 12 637 51 76

Fig. 1S. The comparison of monthly increments of Pb bioaccumulation in various tissues fish of experimental groups 2-5 in the following exposure periods 3, 6, 12, 15, 18 and 24 months. (prox – proximal intestine, distal – distal intestine). Note the different y-axis scale.

Fig. 2S. The comparison of Pb depuration month rates [mg kg^-1^] in studied tissues of Prussian carp females, of four groups of earlier Pb dietary exposure, in dependence on the period of purification (3, 6 or 12 months). Note the different y-axis scale.

**Table 1S.** The significance of the effect of lead dose during exposure on its concentration in individual tissues presented as the Spearman’s correlation coefficients R. *p<0.05, **p<0.01, ***p<0,001.

|  | **Months of experiment** | **kidney** | **gills** | **liver** | **muscle** | **proximal intestine** | **distal intestine** | **skin** | **bone** | **scales** |
| --- | --- | --- | --- | --- | --- | --- | --- | --- | --- | --- |
| exposure to Pb | 3 | 0,61** | 0,66*** | 0,78*** | 0,56** | 0,84*** | 0,85*** | -0,13 | 0,76*** | 0,63*** |
|  | 6 | 0,75*** | 0,87*** | 0,85*** | 0,47* | 0,95*** | 0,78*** | 0,05 | 0,88*** | 0,93*** |
|  | 12 | 0,78*** | 0,89*** | 0,88*** | 0,48* | 0,94*** | 0,68** | 0,43 | 0,85*** | 0,87*** |
|  | 15 | 0,81*** | 0,80*** | 0,89*** | 0,68*** | 0,71*** | 0,76*** | -0,45 | 0,95*** | 0,91*** |
|  | 18 | 0,76*** | 0,85*** | 0,91*** | 0,74*** | 0,73*** | 0,73*** | -0,007 | 0,90*** | 0,94*** |
|  | 24 | 0,58*** | 0,81*** | 0,95*** | 0,87*** | 0,73*** | 0,82*** | 0,1 | 0,94*** | 0,95*** |
| depuration | 3 | 0,30* | 0,74*** | 0,87*** | 0,39** | 0,48* | 0,41* | -0,42 | 0,87*** | 0,96*** |
|  | 6 | 0,25 | 0,36* | 0,75*** | 0,27 | 0,46 | 0,57* | -0,29 | 0,90*** | 0,82*** |
|  | 12 | 0,12 | 0,71*** | 0,77*** | 0,19 | 0,57* | 0,41** | -0,27 | 0,78*** | 0,96*** |
